# Associations of mid-childhood per- and polyfluoroalkyl substances and early childhood metals with mid-childhood antibody titers

**DOI:** 10.1097/EE9.0000000000000471

**Published:** 2026-03-13

**Authors:** Anna Smith, Pi-I D. Lin, Sheryl L. Rifas-Shiman, Diane R. Gold, Abby F. Fleisch, Marie-France Hivert, Emily Oken, Andres Cardenas

**Affiliations:** a Department of Epidemiology and Population Health, Stanford University, Stanford, California; b Division of Chronic Disease Research across the Lifecourse, Department of Population Medicine, Harvard Medical School, Harvard Pilgrim Health Care Institute, Boston, Massachusetts; c Channing Division of Network Medicine, Department of Medicine, Harvard Medical School, Brigham and Women’s Hospital, Boston, Massachusetts; d Department of Environmental Health, Harvard T.H. Chan School of Public Health, Boston, Massachusetts; e Center for Interdisciplinary Population and Health Research, Maine Health Institute for Research, Westbrook, Maine; f Pediatric Endocrinology and Diabetes, Maine Medical Center, Portland, Maine; g Diabetes Unit, Massachusetts General Hospital, Boston, Massachusetts; h Department of Pediatrics, Stanford University, Stanford, California

**Keywords:** Antibody titers, Children’s environmental health, Environmental mixtures metals, PFAS

## Abstract

**Background:**

Per- and polyfluoroalkyl substances (PFAS) and nonessential metals impair immune responses, while essential metals promote immune maintenance. We evaluated associations of childhood PFAS and metal mixtures with measles, mumps, rubella (MMR), pertussis, diphtheria, and tetanus antibody titers.

**Methods:**

We measured mid-childhood (age 7.7 years, interquartile range 7.4, 8.4) plasma PFAS (perfluorooctanoate, perfluorooctane sulfonate, perfluorodecanoate, perfluorohexane sulfonate, 2-(N-methyl-perfluorooctane sulfonamide) acetate, and perfluorononanoate) and early childhood (3.2 years, interquartile range 3.0, 3.5) blood levels of nonessential (arsenic, barium, cadmium, cesium, lead, mercury, strontium, and tin), and essential (cobalt, copper, magnesium, manganese, molybdenum, selenium, and zinc) metals in children from the Project Viva cohort, recruited prenatally between 1999 and 2000 in Massachusetts, United States. We measured plasma MMR, pertussis, diphtheria, and tetanus titers, and the analytical sample of children with all measurements ranged from n = 493–507 for PFAS and n = 179–185 for metals analyses. We used adjusted quantile g-computation and regression models to estimate mixture- and individual PFAS or metal associations, respectively.

**Results:**

Approximately 48% of the children were female, and children received their last MMR or diphtheria, tetanus, pertussis vaccination dose about 3 years before blood antibody titer collection. Contrary to our hypothesis, a one-quartile increase in the mid-childhood PFAS mixture was associated with higher mid-childhood measles [β = 0.06 antibody (Ab) index, 95% confidence interval (CI): 0.02, 0.1], rubella (β = 2.6 IU/mL, 95% CI: 0.5, 4.8), pertussis (log-β = 0.2 IU/mL, 95% CI: 0.2, 0.5), and tetanus (log-β = 0.2 IU/mL, 95% CI: 0.04, 0.4) antibody titers. A one-quartile increase in the early childhood essential metals mixture was associated with lower mid-childhood rubella antibody titers (β = −4.9 IU/mL, 95% CI: −9.0, −0.8). Individual PFAS and metals were associated with pertussis, diphtheria, and tetanus antibody titers in directions contrary to our initial hypotheses.

**Conclusions:**

Our results suggest that mid-childhood blood PFAS and early childhood metals may influence antibody titers, although additional prospective studies are needed.

**What this study adds**

Prior studies found associations between per- and polyfluoroalkyl substances (PFAS) and metals and altered vaccine-induced antibody titer levels. We aimed to expand on the literature by examining associations between mixtures of PFAS and metals and multiple vaccine-induced antibody titer outcomes. We harnessed a large prospective cohort study to examine mixtures of PFAS and metals and multiple vaccine-induced antibody titer outcomes. The study contributes to Environmental Epidemiology’s mission of using novel statistical methods to study mixtures of PFAS and metals relevant to public health in a large cohort study. The results could further guide the understanding of how childhood PFAS and metals impact childhood immune response.

## Introduction

Childhood environmental exposures may modulate immune function, especially during periods of rapid development.^1,2^ Per- and polyfluoroalkyl substances (PFAS) are a family of ubiquitous, stable, synthetic chemicals, and human exposure is associated with immune dysfunction.^3^ Children are exposed through drinking water, food items, indoor dust, air, and household products, and PFAS are widely detected in pregnant persons and children, likely from consumer products.^4^ PFAS induce inflammation, alter cytokine production, interfere with antigens, and modulate cell populations involved in antibody response.^1,5^ Similarly, nonessential metals, such as arsenic and lead, are ubiquitous in the environment, and children are exposed through inhalation of airborne pollutants, soil contact, drinking water, and dietary sources.^6^ Some nonessential metals, such as arsenic and lead, are also toxic metals and harmful at any level in the body, while others, such as strontium and tin, are not required for biological function, although present in the body at trace amounts.^7^ Nonessential metals impact the developing immune system by activating inflammatory pathways and altering cytokine levels.^2,5,8–10^ Together, PFAS and nonessential metals have several key characteristics of immunotoxic agents.^1,2,11^ Essential metals, such as zinc, are antioxidants necessary for immune cell growth and maintenance, including those involved in vaccine response.^12,13^ While evidence exists for the importance of zinc in immune function, there is less research on metals such as manganese and copper. Although the current experimental evidence suggests that certain essential metals are involved in immune function via multiple proposed molecular mechanisms,^14^ additional epidemiological studies are needed.

After routine childhood vaccination, some children will have primary (lack of seroconversion) or secondary (waning immunity) vaccine failure, leaving some children vulnerable to preventable disease and breakthrough infections in settings of low vaccine uptake and limited herd immunity.^15–18^ Environmental chemicals are increasingly recognized as factors that suppress the human immune system leading to primary vaccine failure through immune suppression and reduced antibody production, and secondary vaccine failure due to their potential impact on memory cells.^19,20^ Prior studies found associations between childhood PFAS and metals and childhood vaccine-induced antibody production.^21–32^ A systematic review summarizing the state of the evidence on associations between PFAS and vaccine-induced antibody titers identified common association trends, including high certainty of evidence of inverse associations between childhood perfluorooctanoate (PFOA) and diphtheria and tetanus antibodies, as well as childhood and adult perfluorooctane sulfonate (PFOS) and rubella antibody titers.^23^ Studies thus far have mostly focused on examining diphtheria and tetanus antibody titers in relation to PFAS exposure. In addition, while there is evidence of immune modulation by arsenic and lead in both toxicological and human epidemiological studies, there is mostly some toxicological data with other metals and limited human evidence. In addition, most prior studies examined PFAS and metals individually and not as mixtures.

Thus, we aimed to expand on current literature by conducting mixture analyses and including a broader range of exposures and titers in our analyses. We leveraged a large, prospective prebirth cohort from the United States to examine associations of a mid-childhood PFAS mixture and early childhood metal mixtures with mid-childhood measles, mumps, rubella (MMR) and diphtheria, tetanus, pertussis (DTaP) antibody titers. We hypothesized that the childhood blood PFAS and nonessential metals would be associated with lower mid-childhood antibody titers, while the early childhood essential metals would be associated with higher antibody titers, which could be clinically relevant in the context of reduced immunization and continued disease outbreaks in the United States.

## Methods

### Study population

We recruited pregnant persons from eastern Massachusetts to the prospective prebirth cohort study, Project Viva, between 1999 and 2002, during their first prenatal visit.^33^ Of the 2128 mother-child pairs, 28 participants enrolled more than once due to repeat pregnancies, and we used data from the first completed pregnancy (n = 2100).

Of the child participants from the mother-child pairs, 643 children had complete mid-childhood blood PFAS measurements and 639 of these children had at least one mid-childhood antibody titer measured. Of these children, 598 had medical record data. Of the remaining children with medical record data, 507 and 493, respectively, had medical record confirmed two-dose MMR and five-dose DTaP vaccination at the recommended time, based on US Centers for Disease Control and Prevention (CDC) 2007 recommendations (Table S1; https://links.lww.com/EE/A416) and greater than 14-day difference between vaccine dose and antibody titer blood draw (Figure S1; https://links.lww.com/EE/A416).

Of the child participants from the mother-child pairs, 390 children had complete early childhood blood metal measurements and 233 of these children had at least one mid-childhood antibody titer measured. Of these children, 218 had medical record data. Of the remaining children with medical record data, 185 and 179, respectively, had medical record confirmed two-dose MMR and five-dose DTaP vaccination at the recommended time, based on CDC 2007 recommendations (Table S1; https://links.lww.com/EE/A416) and greater than 14-day difference between vaccine dose and antibody titer blood draw (Figure S2; https://links.lww.com/EE/A416). The characteristics of the participants included in our analytical samples are similar to those of the entire Project Viva cohort (Table 1).^33^

**Table 1.** Characteristics of mother-child pairs from the Project Viva cohort with mid-childhood PFAS or early childhood metal measurements, at least one antibody titer outcome, and fully vaccinated on schedule with the MMR or DTaP vaccine

Demographic characteristics		PFAS and completed MMR series	PFAS and completed DTaP series	Metals and completed MMR series	Metals and completed DTaP series
		n (%) or median (IQR)	n (%) or median (IQR)	n (%) or median (IQR)	n (%) or median (IQR)
Overall		507	493	185	179
Maternal age at enrollment (years)		32.4 (24.4, 35.9)	32.4 (28.2, 35.9)	32.9 (29.7, 36.2)	32.9 (29.7, 36.3)
Maternal race or ethnicity					
	Asian	21 (4.1)	21 (4.3)	5 (2.7)	5 (2.8)
	Black	109 (21.5)	105 (21.3)	31 (16.8)	29 (16.2)
	Hispanic	35 (6.9)	35 (7.1)	7 (3.8)	7 (3.9)
	White	312 (61.5)	303 (61.5)	135 (73.0)	131 (73.2)
	More than one^a^	26 (5.1)	27 (5.5)	7 (3.8)	7 (3.9)
	No self-report	4 (0.8)	2 (0.4)	NA	NA
Maternal education					
	College graduate	316 (62.3)	304 (61.7)	131 (70.8)	125 (69.8)
	Not a college graduate	187 (36.9)	187 (37.9)	54 (29.2)	54 (30.2)
	No self-report	4 (0.8)	2 (0.4)	NA	NA
Child assigned sex at birth					
	Female	237 (46.7)	234 (47.5)	91 (49.2)	87 (48.6)
	Male	270 (53.3)	259 (52.5)	94 (50.8)	92 (51.4)
Child age titer blood draw (years)		7.7 (7.4, 8.4)	7.7 (7.4, 8.3)	7.6 (7.3, 8.0)	7.6 (7.3, 8.0)
Child age last vaccine dose (years)		4.3 (4.1, 5.0)	4.3 (4.1, 5.0)	4.2 (4.1, 5.1)	4.2 (4.1, 5.0)
Child time since last vaccine (years)		3.3 (2.7, 4.0)	3.3 (2.7, 3.9)	3.1 (2.7, 3.7)	3.1 (2.7, 3.7)

a Includes American Indian or Alaskan Native, Asian or Pacific Islander, Black or African American, Hispanic or Latina, White or Caucasian, and Other, Specify, as written in the self-report survey.

Mothers provided written informed consent at enrollment and postnatal follow-up, and children provided verbal assent at mid-childhood. The Institutional Review Board of Harvard Pilgrim Health Care reviewed and approved all study protocols.

### Exposure assessment

We described methods for quantifying mid-childhood (median age = 7.7 years) plasma PFAS in prior studies.^34,35^ Briefly, the Division of Laboratory Sciences at the CDC (Atlanta, Georgia) measured plasma concentrations of PFOS, PFOA, perfluorohexane sulfonate (PFHxS), perfluorononanoate (PFNA), perfluorodecanoate (PFDA), and 2-(N-methyl-perfluorooctane sulfonamide) acetate (MeFOSAA) using online solid-phase extraction combined with isotope dilution high-performance liquid chromatography-tandem mass spectrometry. The limit of detection (LOD) and detection frequency (>60%) for each PFAS are shown in Tables S2 and S3; https://links.lww.com/EE/A416. We assigned concentrations below the LOD the value LOD/(square root of 2). We report results as plasma PFAS concentrations (ng PFAS/mL serum).

We described methods for quantifying early childhood (median age = 3.2 years) red blood cell (RBC) metals in prior studies.^36,37^ Briefly, we measured the concentration of eight nonessential metals (arsenic, barium, cadmium, cesium, lead, mercury, strontium, and tin) and seven essential metals (cobalt, copper, magnesium, manganese, molybdenum, selenium, and zinc) using triple quadrupole inductively coupled plasma mass spectrometry (Agilent 8900; Agilent Technologies, Santa Clara, CA). The LOD and detection frequency (>60%) for each metal are shown in Tables S2 and S3; https://links.lww.com/EE/A416. We assigned concentrations below the LOD the value LOD/(square root of 2). We report results as RBC metal concentrations (ng metal/g RBC).

### Vaccine titers

We stored childhood (median age = 7.7 years) heparin plasma and transported it on dry ice to Boston Children’s Hospital for analysis. We thawed samples and vortexed and centrifuged samples before manual testing. We analyzed MMR and DTaP antibody titers in duplicate following assay insert instructions. Assays included the Measles IgG ELISA Kit (Abnova, Taipei City, Taiwan), Mumps IgG ELISA Kit (Abnova, Taipei City, Taiwan), Rubella virus IgG ELISA Kit (Abnova, Taipei City, Taiwan), Bordetella pertussis Toxin IgG ELISA Kit (Abnova, Taipei City, Taiwan), Human Anti-Diphtheria Toxoid IgG ELISA Assay (XpressBio, Frederick, Maryland), and the Human Anti-Tetanus Toxoid IgG ELISA Assay (XpressBio, Frederick, Maryland). Antibody titers for each assay were reported continuously and categorically, and a summary of the antibody assays and seroprotective cutoffs is detailed in Table S4; https://links.lww.com/EE/A416.

### Covariate assessment

We included the assigned child sex at birth, abstracted from delivery records, as a covariate in main models. There is evidence from prior studies that time since immunization has a strong influence on antibody levels,^18,38^ and so we adjusted for time since most recent immunization (dose number two for MMR analyses and dose number five for DTaP analyses).

Since we restricted our study sample to fully vaccinated participants, we did not adjust for maternal age at enrollment, education (proxy for socioeconomic status), and race and ethnicity, which are associated with blood PFAS, metals, and number of vaccine doses in prior studies,^4,39–41^ although we report these characteristics for study generalizability assessment (Table 1).

### Statistical analysis

We used adjusted quantile g-computation with linear regression models^42^ to estimate the overall association between the mid-childhood PFAS mixture and early childhood metal mixtures and each antibody titer outcome. Quantile g-computation is a parametric, generalized linear model-based implementation of g-computation,^42^ that estimates parameters of a marginal structure model that characterize the change in expected antibody titer outcomes given a joint intervention of all PFAS, conditional on confounders. The estimate is interpreted as associations in each outcome for a one-quantile simultaneous increase in all PFAS or metals in the mixture, referred to as the PFAS or metal mixture exposure-response.^42^

For comparison with prior studies, we conducted linear regression analyses to estimate associations between individual mid-childhood PFAS and early childhood metals with continuous mid-childhood antibody titers, adjusting for covariates. The MMR outcomes are interpreted as the estimated change in antibody titer outcome for a one-unit (ng/mL or ng/g) change in PFAS or metal concentration. For the DTaP models, we log-transformed the titer outcomes to meet model assumptions of normally distributed residuals, and estimates and confidence intervals (CI) were converted using the formulas (e^β^−1) × 100 and (e^95% CI upper or lower bound^−1) × 100^44^ and interpreted as the relative change in median antibody titer [%change (95% CI)] [Ab (antibody) index or IU/mL] when adding one unit of PFAS or metal concentration (ng/mL or ng/g).^43^ We converted 95% CIs to evaluate magnitude of associations and statistical significance.

We considered examining the outcomes categorically, and we refrained for several reasons: (1) in the general population, two age-appropriate MMR vaccine doses are considered evidence of immunity,^44,45^ (2) antibody titers are not the only mechanisms of immunity postvaccine (i.e., cell-based immunity),^44,46^ (3) protective threshold varies by assay and is not universally defined,^47^ except for diphtheria and tetanus in which all our participants were considered above the threshold.^48^ Our study focused on examining how PFAS and metals may impact immunity, and we refrain from making assumptions about whether given antibody titer levels are sufficient to prevent disease in our cohort.

## Results

### Baseline characteristics

We report baseline characteristics for our PFAS, MMR, and DTaP analytical samples in Table 1. In our MMR analytical sample (n = 507), median [interquartile range (IQR)] age of participants was 32.4 years (24.4, 35.9). Most maternal participants self-identified their race as White (61.5%) and received a college education (62.3%). There were more male (53.3%) children than female (46.7%) children. Distributions of demographic characteristics were nearly identical in the DTaP analytical sample (n = 493). Median (IQR) child age at blood draw was 3.3 years (2.7, 4.0), and the median (IQR) age at which the children received their most recent MMR (dose number 2) and DTaP (dose number 5) vaccinations was 4.3 years (4.1, 5.0).

We also report baseline characteristics for our metals, MMR, and DTaP analytical samples in Table 1, which are similar to the PFAS study samples. In our MMR analytical sample (n = 185), median (IQR) age of participants was 32.9 years (29.7, 36.2). Most maternal participants self-identified their race as White (73.0%) and received a college education. There were more male (51.4%) children than female (48.6%) children. Distributions of demographic characteristics were nearly identical in the DTaP analytical sample (n = 179). Median (IQR) child age at blood draw was 7.6 years (7.3, 8.0), and the median (IQR) age at which the children received their most recent MMR (dose number 2) and DTaP (dose number 5) vaccinations was 4.2 years (4.1, 5.1).

### Exposure and outcome distributions

We present mid-childhood PFAS and early childhood metal distributions for each of the respective analytical samples (Table S2 and S3; https://links.lww.com/EE/A416) and Spearman correlation coefficients among the mid-childhood PFAS (Figure S3; https://links.lww.com/EE/A416) and early childhood metals (Figure S4; https://links.lww.com/EE/A416) for each of the analytical samples. Mid-childhood PFAS were mostly moderately correlated, while early childhood metals were mostly mildly correlated.

### Mid-childhood antibody titer distributions

**Figure 1.**
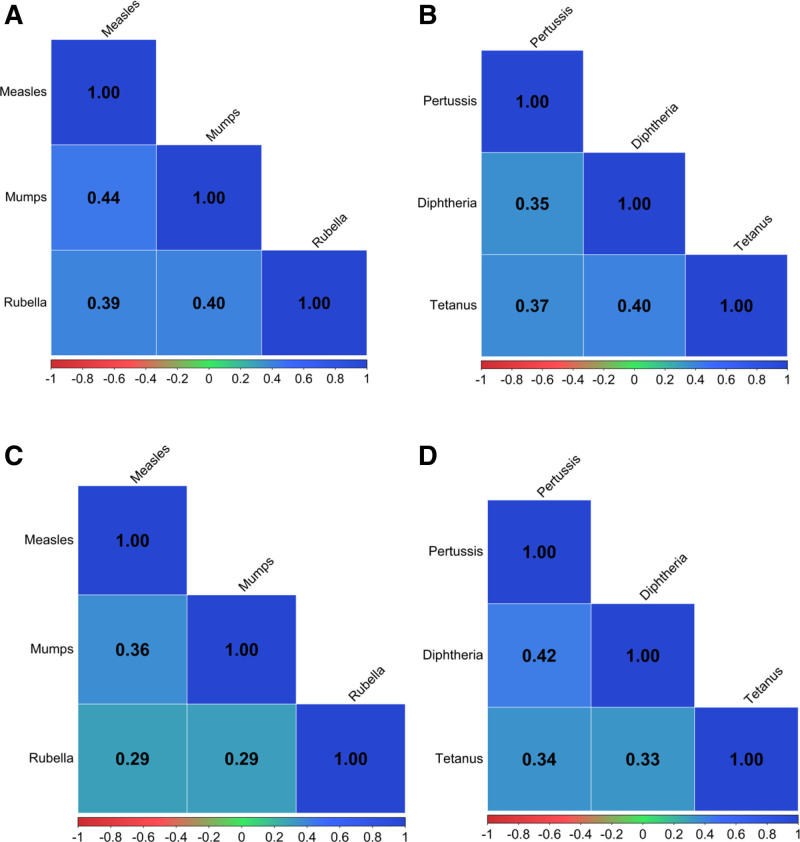
Spearman correlation coefficients among continuous antibody titer outcomes in participants with at least one antibody titer outcome, and (A) PFAS and a complete MMR (n = 507), (B) PFAS and a complete DTaP vaccine series (n = 493), (C) metals and a complete MMR (n = 185), and (D) metals and a complete DTaP vaccine series (n = 179).

We show Spearman correlation coefficients among the antibody titers for each of the analytical samples in Figure 1. The outcomes were positively and moderately correlated, with the strongest correlation between measles and mumps (*r* = 0.44 and 0.36 for PFAS and metal samples, respectively). We present the antibody titer distributions for the PFAS and metals analytical samples in Table 2, and the categorical distributions in Table S5; https://links.lww.com/EE/A416 for reference.

**Table 2.** Distributions of antibody titers in mid-childhood blood for participants with mid-childhood PFAS or early childhood metals measurements, at least one antibody titer outcome, and fully vaccinated on schedule with the MMR or DTaP vaccine

Exposure	Antibody titers	Total n	Median (IQR) (Ab index^a^ or IU/mL^b^)	Minimum	Maximum
PFAS	Measles	507	1.1 (0.9, 1.3)	0.1	2.0
	Mumps	507	2.9 (2.2, 3.5)	0.3	5.6
	Rubella	489	33.9 (21.4, 46.9)	3.0	110.0
	Pertussis	484	6.3 (3.0, 13.5)	−0.09	2200.0
	Diphtheria	474	0.5 (0.3, 0.8)	0.07	4.0
	Tetanus	446	1.0 (0.4, 2.1)	0.03	39.0
Metals	Measles	185	1.1 (0.9, 1.3)	0.3	1.9
	Mumps	185	2.9 (2.1, 3.6)	0.6	5.2
	Rubella	176	33.4 (20.7, 47.4)	5.1	81.0
	Pertussis	175	6.9 (3.4, 13.7)	0.4	110.0
	Diphtheria	168	0.5 (0.29, 0.8)	0.1	4.0
	Tetanus	163	1.0 (0.5, 2.1)	0.04	39.0

a Measles and mumps units are Ab index.

b Rubella, pertussis, diphtheria, and tetanus units are IU/mL.

Ab, antibody; n, sample size.

### Associations of per- and polyfluoroalkyl substances and metals with antibody titers

We present mixture analysis results in Table 3. Contrary to our hypothesis, a one-quartile increase in the mid-childhood PFAS mixture was associated with higher mid-childhood measles [β = 0.06 Ab index, 95% CI: 0.02, 0.1], rubella (β = 2.6 IU/mL, 95% CI: 0.5, 4.8), pertussis (log-β = 0.2 IU/mL, 95% CI: 0.2, 0.5), and tetanus (log-β = 0.2 IU/mL, 95% CI: 0.04, 0.4) antibody titers. A one-quartile increase in the early childhood essential metals mixture was associated with lower mid-childhood rubella antibody titers (β = −4.9 IU/mL, 95% CI: −9.0, −0.8). The nonessential metals mixture was not significantly associated with any of the antibody titer outcomes.

**Table 3.** Quantile g-computation exposure-response estimates (95% bootstrap confidence intervals) for associations of the mid-childhood PFAS mixture (ng/mL), nonessential metals mixture (ng/g), or essential metals mixture (ng/g) and measles, mumps (Ab index), rubella, pertussis, diphtheria, and tetanus (IU/mL) antibody titers

Mixture	Antibody titer (n)	β (95% bootstrap CI)^a^,^b^
PFAS (MeFOSSA, PFDA, PFHxS, PFNA, PFOA, and PFOS)	Measles (n = 507)	0.06 (0.02, 0.1)
	Mumps (n = 507)	0.1 (−0.02, 0.2)
	Rubella (n = 489)	2.6 (0.5, 4.8)
	Pertussis (n = 484)	0.2 (0.2, 0.5)
	Diphtheria (n = 474)	0.1 (−0.03, 0.2)
	Tetanus (n = 446)	0.2 (0.04, 0.4)
Nonessential metals (Arsenic, Barium, Cadmium, Cesium, Lead, Mercury, Strontium, and Tin)	Measles (n = 185)	0.01 (−0.1, 0.1)
	Mumps (n = 185)	−0.1 (−0.5, 0.2)
	Rubella (n = 176)	5.2 (−0.8, 11.2)
	Pertussis (n = 175)	0.2 (−0.2, 0.6)
	Diphtheria (n = 168)	0.1 (−0.1, 0.4)
	Tetanus (n = 163)	0.3 (−0.2, 0.7)
Essential metals (Cobalt, Copper, Magnesium, Manganese, Molybdenum, Selenium, and Zinc)	Measles (n = 185)	−0.05 (−0.1, 0.03)
	Mumps (n = 185)	−0.1 (−0.4, 0.2)
	Rubella (n = 176)	−4.9 (−9.0, −0.8)
	Pertussis (n = 175)	−0.2 (−0.5, 0.1)
	Diphtheria (n = 168)	0.1 (−0.1, 0.2)
	Tetanus (n = 163)	0.1 (−0.3, 0.4)

a Interpretation for measles, mumps, and rubella: difference in each outcome for one-quartile increase in all PFAS, nonessential, or essential metals, conditional on the covariates assigned sex at birth, time since last vaccination, and metals not included in the mixture (metal analyses only).

b Interpretation for pertussis, diphtheria, and tetanus: difference in each log-outcome for one-quartile increase in all PFAS, nonessential, or essential metals, conditional on the covariates assigned sex at birth, time since last vaccination, and metals not included in the mixture (metal analyses only).

Ab, antibody; PFDA, perfluorodecanoic acid.

We present individual analysis results for measles, mumps, and rubella in Table 4. We did not observe statistically significant associations between the PFAS or metals and measles, mumps, or rubella antibody titers.

**Table 4.** Associations of mid-childhood blood PFAS (ng/mL) and early childhood metals (ng/g) and measles (n = 507 PFAS; n = 185 metals), mumps (n = 507 PFAS; n = 185 metals) (Ab index), and rubella (n = 489 PFAS; n = 176 metals) (IU/mL), antibody titers

PFAS or metal	Measles β (95% CI)^a^,^b^	Mumps β (95% CI)^a^,^b^	Rubella β (95% CI)^a^,^b^
MeFOSSA	−0.01 (−0.05, 0.03)	−0.08 (−0.2, 0.04)	−0.2 (−2.2, 1.8)
PFDA	0.1 (−0.002, 0.3)	0.2 (−0.2, 0.6)	5.9 (−1.3, 13.1)
PFHxS	−0.001 (−0.01, 0.003)	0.001 (−0.01, 0.01)	−0.1 (−0.3, 0.1)
PFNA	0.001 (−0.01, 0.01)	0.02 (−0.01, 0.06)	0.2 (−0.5, 0.8)
PFOA	0.01 (−0.01, 0.02)	0.01 (−0.02, 0.05)	0.3 (−0.4, 0.9)
PFOS	0.003 (−0.002, 0.01)	0.004 (−0.01, 0.02)	0.05 (−0.2, 0.3)
Arsenic	0.01 (−0.02, 0.05)	0.03 (−0.1, 0.1)	1.7 (−0.2, 3.7)
Barium	0.01 (−0.02, 0.03)	0.0 (−0.1, 0.1)	0.2 (−0.9, 1.3)
Cadmium	0.03 (−0.03, 0.09)	0.1 (−0.1, 0.3)	0.8 (−2.3, 3.9)
Cesium	−0.01 (−0.1, 0.1)	0.1 (−0.2, 0.4)	0.8 (−2.3, 3.9)
Lead	0.0 (−0.05, 0.05)	−0.1 (−0.3, 0.05)	0.3 (−2.5, 3.2)
Mercury	0.02 (−0.0, 0.05)	0.02 (−0.1, 0.1)	0.3 (−1.0, 1.7)
Strontium	0.0 (−0.1, 0.1)	−0.2 (−0.5, 0.01)	3.8 (−0.2, 7.8)
Tin	−0.0 (−0.1, 0.02)	−0.1 (−0.2, 0.04)	−1.2 (−3.5, 1.0)
Cobalt	−0.01 (−0.1, 0.03)	0.05 (−0.1, 0.2)	−2.2 (−4.6, 0.2)
Copper	0.04 (−0.2, 0.2)	−0.1 (−0.7, 0.6)	−3.5 (−14.0, 6.9)
Magnesium	−0.1 (−0.3, 0.1)	−0.1 (−0.7, 0.6)	−10.0 (−21.2, 1.1)
Manganese	−0.1 (−0.1, 0.05)	−0.2 (−0.6, 0.1)	−4.9 (−10.1, 0.4)
Molybdenium	−0.02 (−0.1, 0.04)	−0.1 (−0.3, 0.1)	1.1 (−2.2, 4.5)
Selenium	0.01 (−0.1, 0.2)	−0.1 (−0.6, 0.5)	−2.3 (−11.4, 6.8)
Zinc	0.1 (−0.1, 0.2)	0.2 (−0.4, 0.7)	−2.4 (−11.4, 6.7)

a Models adjusted for assigned child sex at birth and time since last vaccination. Estimates interpreted as the estimated change in antibody titer outcome (Ab index or IU/mL) for a one unit (ng/mL or ng/g) change in PFAS or metal concentration.

b For metals, interpret as per doubling in exposure, since metals are log2-transformed.

Ab, antibody.

We present individual analysis results for pertussis, diphtheria, and tetanus in Table 5. A one ng/mL increment in PFDA, PFHxS, PFOA, and PFOS was associated with 202.9% (95% CI: 73.5, 428.9), 2.3% (95% CI: 0.4, 4.2), 8.1% (95% CI: 2.9, 13.6), and 3.5% (95% CI: 1.5, 5.6) higher pertussis antibody titers. A one ng/mL increment in MeFOSAA, PFDA, PFOA, and PFOS was associated with 17.8% (95% CI: 0.02, 38.7), 139.7% (95% CI: 41.9, 305.2), 8.9% (95% CI: 3.9, 14.2), and 2.7% (95% CI: 0.8, 4.6) higher tetanus antibody titers.

**Table 5.** Associations of mid-childhood blood PFAS (ng/mL) and early childhood metals (ng/g) and pertussis (n = 483 PFAS; n = 175 metals), diphtheria (n = 474 PFAS; n = 168 metals), and tetanus (n = 446 PFAS; n = 163 metals), antibody titers (IU/mL)

PFAS or metal	Pertussis %Δ (95% CI)^a^	Diphtheria %Δ (95% CI)^a^	Tetanus %Δ (95% CI)^a^
MeFOSAA	15.1 (−1.7, 34.7)	0.1 (−8.1, 9.0)	17.8 (0.02, 38.7)
PFDA	202.9 (73.5, 428.9)	25.5 (−7.3, 69.9)	139.7 (41.9, 305.2)
PFHxS	2.3 (0.4, 4.2)	0.4 (−0.6, 1.4)	1.4 (−0.4, 3.3)
PFNA	0.2 (−4.3, 4.8)	0.9 (−1.6, 3.4)	1.0 (−3.2, 5.3)
PFOA	8.1 (2.9, 13.6)	1.1 (−1.6, 3.9)	8.9 (3.9, 14.2)
PFOS	3.5 (1.5, 5.6)	0.2 (−09, 1.3)	2.7 (0.8, 4.6)
Arsenic	3.9 (−4.6, 13.2)	1.2 (−4.1, 6.9)	1.5 (−7.1, 10.9)
Barium	−0.7 (−1.6, 0.2)	−0.3 (−0.9, 0.3)	0.2 (−0.8, 1.2)
Cadmium	5.7 (−58.8, 171.2)	52.0 (−16.2, 175.8)	62.0 (−38.7, 327.8)
Cesium	−2.0 (−19.6, 19.3)	9.0 (−3.9, 23.5)	−2.9 (−21.1, 19.6)
Lead	0.1 (−0.7, 0.9)	0.6 (0.1, 1.2)	−0.1 (−1.0, 0.7)
Mercury	1.3 (−6.1, 9.3)	−3.1 (−7.6, 1.7)	2.4 (−5.4, 10.8)
Strontium	−0.1 (−7.8, 8.2)	−1.6 (−6.5, 3.6)	1.1 (−6.9, 9.9)
Tin	3.7 (−37.5, 71.8)	−15.9 (−39.6, 17.0)	−1.5 (−42.7, 69.2)
Cobalt	−20.9 (−99.0, 5888.3)	4.4 (−94.0, 1712.4)	−92.9 (−99.9, 640.3)
Copper	−0.2 (−0.4, −0.1)	−0.01 (−0.1, 0.1)	0.1 (−0.1, 0.3)
Magnesium	−0.0 (−0.0, 0.0)	0.0 (−0.0, 0.0)	0.0 (−0.0, 0.0)
Manganese	−1.9 (−4.6, 0.9)	−0.5 (−2.3, 1.3)	1.8 (−1.2, 4.9)
Molybdenium	8.1 (−34.6, 78.9)	2.6 (−27.3, 45.0)	−0.9 (−43.8, 74.9)
Selenium	−0.2 (−0.5, 0.2)	0.1 (−0.2, 0.3)	0.1 (−0.3, 0.5)
Zinc	0.0 (−0.01, 0.02)	0.0 (−0.01, 0.01)	0.01 (−0.01, 0.02)

a Models adjusted for assigned child sex at birth and time since last vaccination. We log-transformed the titer outcomes and estimates and confidence intervals were converted using the formulas (e^β^−1) × 100 and (e^95% CI upper or lower bound^−1) × 100 and interpreted as the relative change in median antibody titer (%Δ [95% CI]) when adding one unit of PFAS or metal concentration.

%Δ, percent change.

In addition, a one ng/mL increment in lead was associated with 0.6% (95% CI: 0.1, 1.2) higher diphtheria antibody titers, while a one ng/mL increment in copper was associated with −0.2% (95% CI: −0.4, −0.1) lower pertussis antibody titers.

## Discussion

In contrast to our initial hypotheses, we observed mid-childhood PFAS mixture associations with higher mid-childhood measles, rubella, pertussis, and tetanus antibody titers, as well as an association between the early childhood essential metal mixture and lower mid-childhood rubella antibody titers. Our individual PFAS and metal associations also contradicted our a priori hypotheses.

In our study, participants had a distribution of titers, and some of the environmental chemicals we assessed were associated with titer levels. Primary or secondary vaccine failure occurs when an individual does not produce antibodies after vaccination or when an initially protective immune response wanes over time,^18^ and could result in breakthrough infections in areas where herd immunity is not achieved.^49^ A recent meta-analysis estimated that overall seroconversion rates, defined as the rate of production of antibodies in the blood of a person who did not previously have them, are 96% for measles, 93% for mumps, and 98% for rubella after one or two doses of the trivalent MMR vaccine.^18^ Absence of detectable antibodies is not necessarily correlated with loss of immunity, since immunity can persist from immunological memory.^50^ There is evidence of limited longevity of protection, particularly to mumps, as people with nondetectable mumps antibodies may have limited circulating memory B cells.^51^ A complete five-dose CDC-recommended DTaP vaccine series^52^ has a clinical efficacy of 100% for tetanus and 97% for diphtheria, while the acellular pertussis component provides protection for about 98% of children after the last dose of the series, although this wanes to about 71% after 5 years.^16^

Contrary to our initial hypotheses, we found a cross-sectional association between the mid-childhood PFAS mixture and higher measles, rubella, pertussis, and tetanus antibody titers. We also found associations between individual PFAS (PFDA, PFHxS, PFOA, and PFOS) and higher pertussis antibody titers and between individual PFAS (MeFOSAA, PFDA, PFOA, and PFOS) and higher tetanus antibody titers. In a cross-sectional and longitudinal study in the Faroe Islands, higher mid-childhood PFDA (age 7 years) was associated with higher mid-childhood tetanus titers in adolescence (age 13 years),^25^ which was consistent with our study findings. However, in contrast to our findings, mid-childhood PFDA in the Faroe Islands study was prospectively associated with lower diphtheria titers in adolescence (age 13 years).^25^ In the same Faroe Islands cohort, early childhood (age 5 years) PFOS and PFOA was associated with a higher odds of having tetanus and diphtheria antibodies below the clinically protective level (age 7 years),^24^ and mid-childhood (age 7 years) PFOA and PFOS were cross-sectionally associated with lower diphtheria antibodies (age 7 years).^27^ Consistent with the Faroe Islands studies, in a longitudinal and cross-sectional study of children (age 7–12 years) from Greenland, PFHxS, PFOS, PFNA, and PFDA were associated with higher odds of not having protective levels of diphtheria antibodies.^32^ In a cross-sectional study of adolescents (ages 12–19 years) from the United States National Health and Nutrition Examination Survey (NHANES), PFOS was associated with reduced mumps and rubella antibody concentrations.^30^ It is unclear what factors may explain the contradictory findings. One factor is that it is possible the timing of exposure matters, since both the exposures and titers were measured at different time points in the studies. Another difference between studies is differing vaccination schedules in countries. For example, while the United States recommended five DTaP vaccines at 2, 6, 12, 15–18 months, and 4–6 years,^53^ the Faroe Islands recommended DT vaccination at 3, 5, 12 months, and 5 years, along with pertussis and polio with the booster.^25^

Findings from studies in adult populations are also inconsistent with our study findings. For example, in a cross-sectional study of adolescents and adults from the United States NHANES, PFOA was associated with lower rubella antibody titers.^28^ In a longitudinal study of adults from Copenhagen, Denmark, PFOS, PFNA, and PFDA was associated with lower diphtheria antibody titers after booster vaccination.^54^ In contrast, there were no longitudinal associations observed between PFOS, PFOA, PFNA, PFDA, or PFHxS and tetanus and diphtheria antibody titers in a population of adults from the Faroe Islands.^29^ In a systematic review and meta-analysis examining associations between PFOA, PFOS, PFHxS, PFNA, and PFDA, there was high certainty of evidence that childhood PFOA is associated with lower rubella and diphtheria antibodies.^23^

Fewer epidemiological studies examining childhood metals and antibody titers exist in the literature for comparison with our findings. In our study, early childhood RBC lead was associated with higher mid-childhood diphtheria antibody titers. In contrast, a study in children from Mexico City found that higher perinatal dentin lead concentration was associated with lower diphtheria antibodies at an average age of 5 years, while higher postpartum dentin lead was associated with lower tetanus antibodies at an average age of 5 years.^22^ These study’s findings are not directly comparable, however, due to the different exposure biological matrices (RBC vs. lead) and different exposure time points (perinatal vs. early childhood). In a cross-sectional study of children (age 6–17 years) from the United States, the odds of having a seronegative anti-measles antibody level was approximately two-fold greater for children with blood lead concentrations between 1 and 5 μg/dL, compared with children with blood lead concentrations <1 μg/dL.^26^ Similarly, it is possible that findings differ due to exposure and outcome timing differences and the cross-sectional versus prospective study designs and additional studies may need to elucidate sensitive periods of development by which metals are associated with antibody titers.

We found an association between the early childhood essential metal mixture and lower rubella antibody titers and with copper and lower pertussis antibody titers. These findings were in contrast to our hypothesis that the essential metals would be associated with higher antibody titers, as metals such as magnesium play a pivotal role in immune regulation and function,^55^ and copper deficiency has been associated with lower antibody titers in experimental studies.^56^ However, in a study of children ages 3–7 years chronically exposed to multiple heavy metals in an e-waste recycling area of Guiyu, China, children in the high blood copper (upper median value) group had significantly higher odds of having lower antibody titers to tetanus and Japanese encephalitis and higher odds of lower diphtheria, pertussis, and hepatitis B antibody titers, although these did not reach statistical significance.^57^ In the study in China, children had higher median plasma copper (919 µg/L exposed group vs. 842 µg/L unexposed group)^57^ versus 561 ng/g RBC copper in the Project Viva cohort, although levels between plasma and RBCs are not directly comparable.

One strength of our study is our inclusion of measles, mumps, rubella, and pertussis antibody titers in our analysis, as most prior literature examined diphtheria and tetanus antibody titers only. We also examined associations between mixtures of PFAS and metals, whereas the majority of prior studies looked at associations with individual chemicals. The PFAS plasma concentrations in Project Viva were similar to those reported in US children in the NHANES during the same period, from 2007 to 2008,^58^ and blood lead levels in Project Viva are comparable to those of the broader United States pediatric population.^37,59^

In addition to our strengths, the study at several limitations, including (1) our analysis of mid-childhood PFAS and mid-childhood antibody titers was cross-sectional, and so we were unable to establish temporality, (2) our study population demographic distributions (relatively high percentage of white, high income, and college educated participants) are not generalizable to the United States or world population, and (3) we chose not to adjust for multiple testing due to the relatedness of the associations (hypotheses are not mutually independent) and prior studies suggesting the global null is unlikely,^60^ and thus it is possible some findings could be due to chance.

Overall, we observed several associations between the childhood PFAS and metals and childhood antibody titers in directions of association that contradicted our a priori hypotheses and prior studies. Our findings highlight the complexity of how early environmental exposures may influence immune system development and processes in childhood. Future studies should continue to examine windows of susceptibility, as well as the molecular mechanisms that could explain our findings.

## Conflicts of interest statement

The authors declare that they have no conflicts of interest with regard to the content of this report.

## ACKNOWLEDGMENTS

*The authors thank the staff and participants of Project Viva.*

## Supplementary Material
