# Supplementary material for: Associations of mid-childhood per- and polyfluoroalkyl substances and early childhood metals with mid-childhood antibody titers
Source: Environ Epidemiol. 2026 Mar 13;10(2):e471. doi: 10.1097/EE9.0000000000000471 (PMC12991553; doi:10.1097/EE9.0000000000000471)
Supplement: Supplementary file 1 [file ee9-10-e471-s001.pdf]

Supplemental Material

Table S1. Recommended vaccination schedule from the year 2007 for persons aged 0-6 years, as detailed by the United States Centers for Disease Control and Prevention (CDC).

	Age						
Vaccine	2 months	4 months	6 months	12 months	15 months	18 months	4-6 years
Diptheria, Tetanus, Pertussis	1st dose	2nd dose	3rd dose		4th dose		5th dose
Measles, Mumps, Rubella				1st dose			2nd dose

Table S2. Distributions of mid-childhood per- and polyfluoroalkyl substances (PFAS) and early childhood metals for participants with all PFAS (*n*=507) and metals (*n*=185) measurements and with at least one MMR vaccine antibody titer measurement.

Metal[a] or PFAS[b]	Median (IQR)	Min	Max	% >LOD	LOD
MeFOSAA (ng/mL)	0.3 (0.1, 0.6)	0.07	6.6	67	0.1
PFDA (ng/mL)	0.3 (0.2, 0.5)	0.07	1.7	89	0.1
PFHxS (ng/mL)	1.9 (1.1, 3.3)	0.07	57.0	100	0.1
PFNA (ng/mL)	1.5 (1.1, 2.3)	0.07	26.0	100	0.1
PFOA (ng/mL)	4.3 (3.1, 5.9)	0.07	14.0	100	0.1
PFOS (ng/mL)	6.1 (4.1, 9.6)	0.07	51.0	100	0.1
Arsenic (ng/g)	0.4 (0.2, 0.6)	0.07	24.0	100	0.05
Barium (ng/g)	0.7 (0.1, 1.9)	0.02	220.0	70	0.17
Cadmium (ng/g)	0.2 (0.1, 0.2)	0.05	2.2	100	0.02
Cesium (ng/g)	2.5 (2.0, 3.1)	1.2	5.5	100	0.01
Lead (ng/g)	26.1 (18.2, 44.1)	6.8	120.0	100	0.17
Mercury (ng/g)	0.5 (0.2, 1.5)	0.04	13.0	99	0.06
Strontium (ng/g)	2.0 (1.7, 2.6)	0.9	20.0	100	0.22
Tin (ng/g)	0.2 (0.2, 0.4)	0.05	2.4	90	0.10
Cobalt (ng/g)	0.0 (0.0, 0.0)	0.1	0.3	68	0.02
Copper (ng/g)	561.0 (524.0, 635.0)	340.0	880.0	100	0.02
Magnesium (ng/g)	44400.0 (40800.9, 48500.0)	24000.0	64000	100	1.64
Manganese (ng/g)	17.9 (14.3, 21.2)	7.6	36.0	100	0.15
Molybdenium (ng/g)	0.3 (0.2, 0.4)	0.09	2.9	100	0.06
Selenium (ng/g)	208.0 (189.0, 240.0)	110.0	450.0	100	0.17
Zinc (ng/g)	6660.0 (6040.0, 7580.0)	4000.0	9800	100	4.29

Abbreviations: IQR, interquartile range; LOD, limit of detection; Max, maximum; MeFOSAA, 2-(N-Methyl-perfluorooctane sulfonamido) acetic acid; Min; minimum; PFAS, per- and polyfluoroalkyl substances; PFDeA, perfluorodecanoic acid; PFHxS, perfluorohexane sulfonate; PFNA, perfluorononanoic acid; PFOA, perfluorooctanoic acid; PFOS, perfluorooctane sulfonate.
[a]Metal concentrations are presented per gram of separated red blood cell.
[b]PFAS concentrations are presented per mL of plasma.

Table S3. Distributions of mid-childhood per- and polyfluoroalkyl substances (PFAS) and early childhood metals for participants with all PFAS (*n*=493) and metals (*n*=179) measurements and with at least one DTaP vaccine antibody titer measurement.

Metal[a] or PFAS[b]	Median (IQR)	Min	Max	% >LOD	LOD
MeFOSAA (ng/mL)	0.3 (0.1, 0.6)	0.07	6.6	67	0.1
PFDA (ng/mL)	0.3 (0.2, 0.5)	0.07	1.7	89	0.1
PFHxS (ng/mL)	1.9 (1.1, 3.3)	0.07	50.0	100	0.1
PFNA (ng/mL)	1.5 (1.1, 2.3)	0.07	26.0	100	0.1
PFOA (ng/mL)	4.4 (3.0, 6.0)	0.07	14.0	100	0.1
PFOS (ng/mL)	6.0 (4.1, 9.5)	0.07	51.0	100	0.1
Arsenic (ng/g)	0.3 (0.2, 0.6)	0.07	24.0	100	0.05
Barium (ng/g)	0.7 (0.1, 1.9)	0.02	220.0	70	0.17
Cadmium (ng/g)	0.2 (0.1, 0.2)	0.05	1.7	100	0.02
Cesium (ng/g)	2.5 (2.0, 3.1)	1.2	5.5	100	0.01
Lead (ng/g)	26.8 (18.8, 44.5)	6.8	120.0	100	0.17
Mercury (ng/g)	0.5 (0.2, 1.5)	0.04	13.0	99	0.06
Strontium (ng/g)	2.1 (1.7, 2.5)	0.9	20.0	100	0.22
Tin (ng/g)	0.2 (0.2, 0.4)	0.04	2.4	90	0.10
Cobalt (ng/g)	0.0 (0.0, 0.1)	0.01	0.3	67	0.02
Copper (ng/g)	559.0 (519.5, 636.5)	340.0	880.0	100	0.02
Magnesium (ng/g)	44600.0 (40800.0, 48650.0)	24000.0	64000	100	1.64
Manganese (ng/g)	17.9 (14.2, 21.1)	7.6	36.0	100	0.15
Molybdenium (ng/g)	0.3 (0.2, 0.4)	0.09	2.9	100	0.06
Selenium (ng/g)	208.0 (189.5, 240.0)	110.0	450.0	100	0.17
Zinc (ng/g)	6660.0 (6020.0, 7555.0)	4000.0	9800	100	4.29

Abbreviations: IQR, interquartile range; LOD, limit of detection; Max, maximum; MeFOSAA, 2-(N-Methyl-perfluorooctane sulfonamido) acetic acid; Min; minimum; PFAS, per- and polyfluoroalkyl substances; PFDeA, perfluorodecanoic acid; PFHxS, perfluorohexane sulfonate; PFNA, perfluorononanoic acid; PFOA, perfluorooctanoic acid; PFOS, perfluorooctane sulfonate.
[a]Metal concentrations are presented per gram of separated red blood cell.
[b]PFAS concentrations are presented per mL of plasma.

Table S4. Summary of antibody assays and cutoffs.[1]

Antibody Titer	Assay	Ab (Antibody) Index Interpretation
Measles	Measles IgG ELISA Kit (Abnova, Taipei City, Taiwan)	<0.9 Ab index, No detectable antibody to Measles IgG by ELISA;<br>0.9-1.1 Ab index, Borderline positive;<br>>1.1 Ab index, Detectable antibody to Measles IgG by ELISA.
Mumps	Mumps IgG ELISA Kit (Abnova, Taipei City, Taiwan)	<0.9 Ab index, No detectable antibody to Mumps IgG by ELISA;<br>0.9-1.1 Ab index, Borderline positive;<br>>1.1 Ab index, Detectable antibody to Mumps IgG by ELISA.
Rubella	Rubella virus IgG ELISA Kit (Abnova, Taipei City, Taiwan)	<10 IU/mL, Negative;<br>10-15 IU/mL, grey zone (equivocal);<br>>15 IU/mL, Positive.
Pertussis	Bordetella pertussis Toxin IgG ELISA Kit (Abnova, Taipei City, Taiwan)	<40 IU/mL, Negative;<br>40-100 IU/mL, borderline;<br>>100 IU/mL, Positive.
Diphtheria	Human Anti-Diptheria Toxoid IgG ELISA Assay (XpressBio, Frederick, MD, USA)	>=0.01 IU/mL, Positive.
Tetanus	Human Anti-Tetanus Toxoid IgG ELISA Assay (XpressBio, Frederick, MD, USA)	>=0.01 IU/mL, Positive.

[1]Seroprotective threshold levels based on assay recommendations and not on universal guidelines.

Table S5. Categorical distributions of antibody titers in mid-childhood blood for participants with mid-childhood PFAS or early childhood metals measurements and covariates.

Exposure	Antibody Titers	Samples Tested (*n*)	Above seroprotective threshold[1] *n* (%)	Borderline[1] *n* (%)	Below seroprotective threshold[1] *n* (%)
PFAS	Measles	507	270 (53.3)	107 (21.1)	130 (25.6)
	Mumps	507	488 (96.3)	7 (1.4)	12 (2.4)
	Rubella	489	429 (87.7)	28 (5.7)	32 (6.5)
	Pertussis	484	7 (1.4)	31 (6.4)	446 (92.1)
	Diphtheria	474	474 (100.0)	0 (0)	0 (0)
	Tetanus	446	446 (100.0)	0 (0)	0 (0)
Metals	Measles	185	98 (53.0)	41 (22.2)	46 (24.9)
	Mumps	185	180 (97.3)	1(0.5)	4 (2.2)
	Rubella	176	151 (85.8)	12 (6.8)	13 (7.4)
	Pertussis	175	1 (0.6)	9 (5.0)	165 (92.2)
	Diphtheria	168	168 (100)	0 (0)	0 (0)
	Tetanus	163	163 (100)	0 (0)	0 (0)

Abbreviations: *n*, sample size; PFAS, per- and polyfluoroalkyl substances.
[1]Based on assay recommendations and not on universal guidelines.

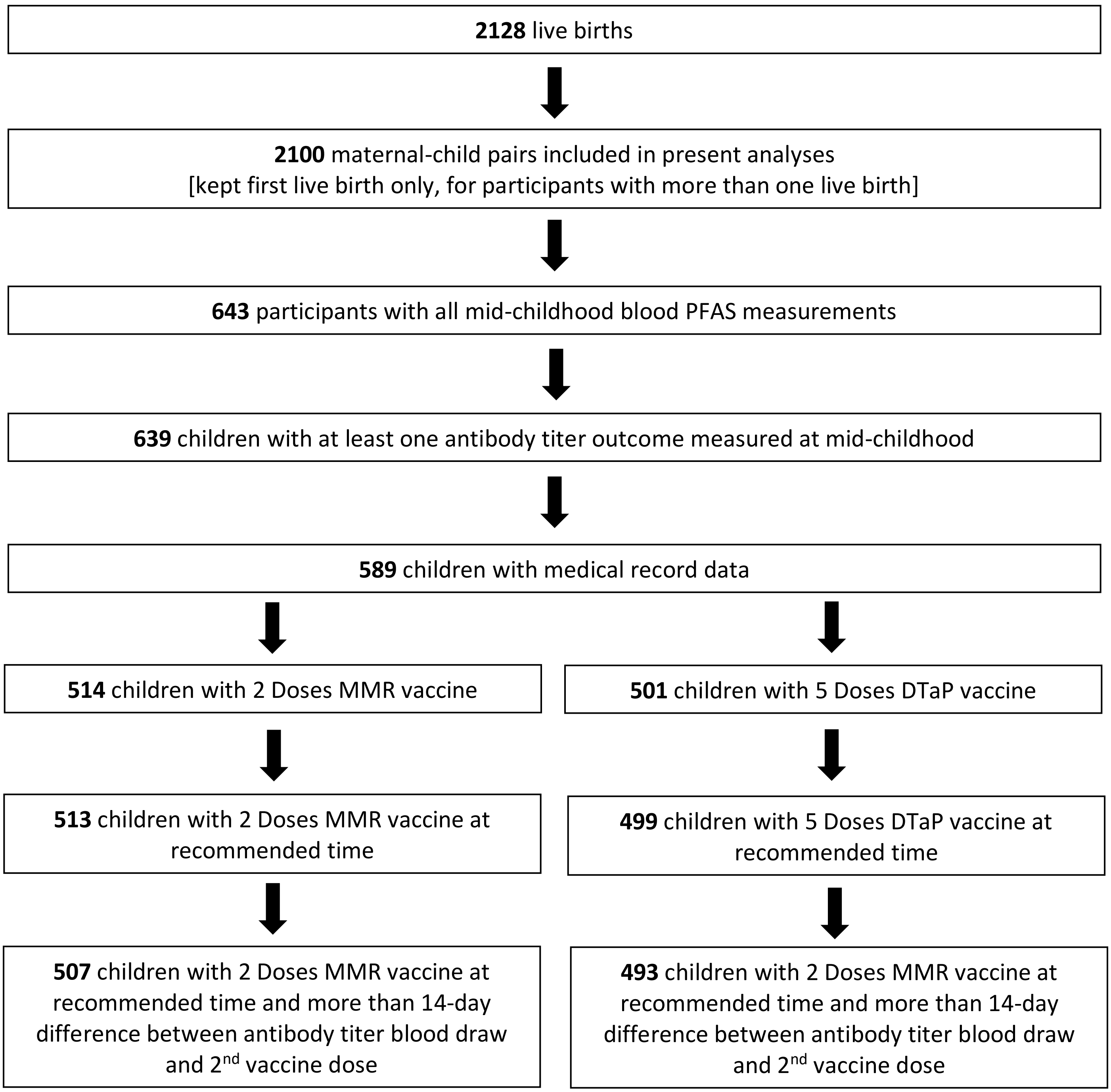

Figure S1. Flow chart of participant inclusion for mid-childhood PFAS and antibody titer analyses.

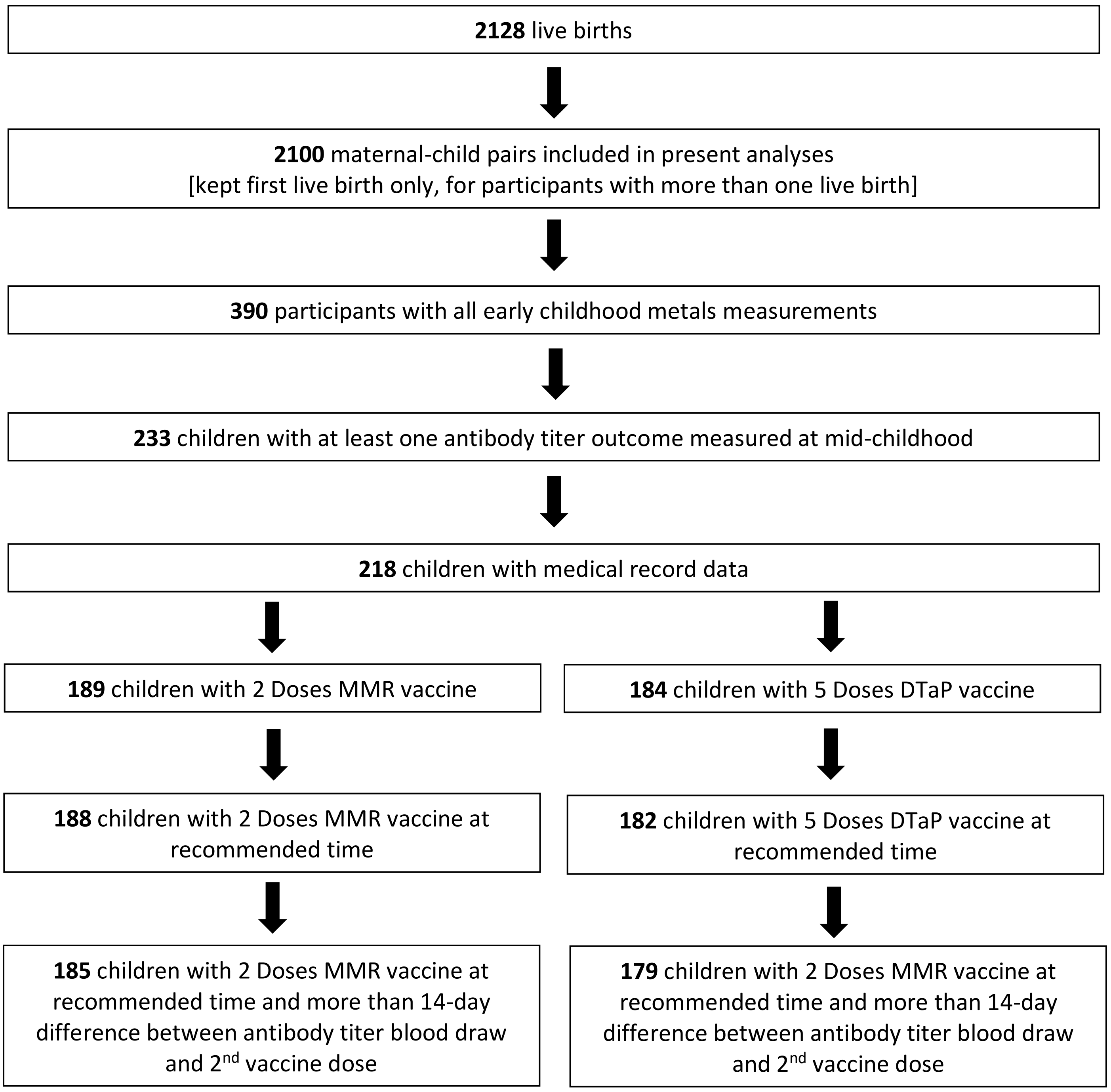

Figure S2. Flow chart of participant inclusion for early childhood metals and antibody titer analyses.

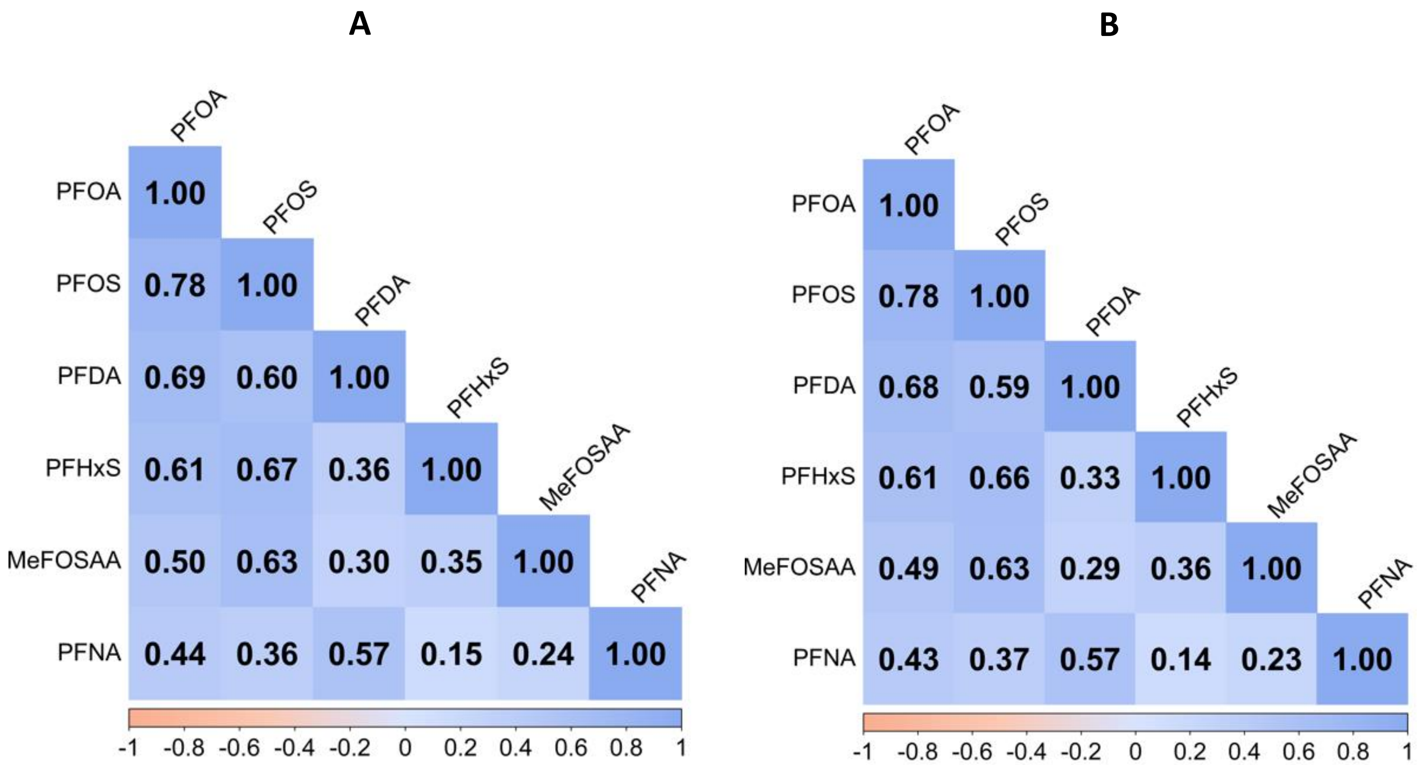

Figure S3. Spearman correlation coefficients among mid-childhood PFAS measurements in participants with at least one antibody titer outcome, and **(A)** a complete MMR (*n*=507) or **(B)** (*n*=493) a complete DTaP vaccine series.

A

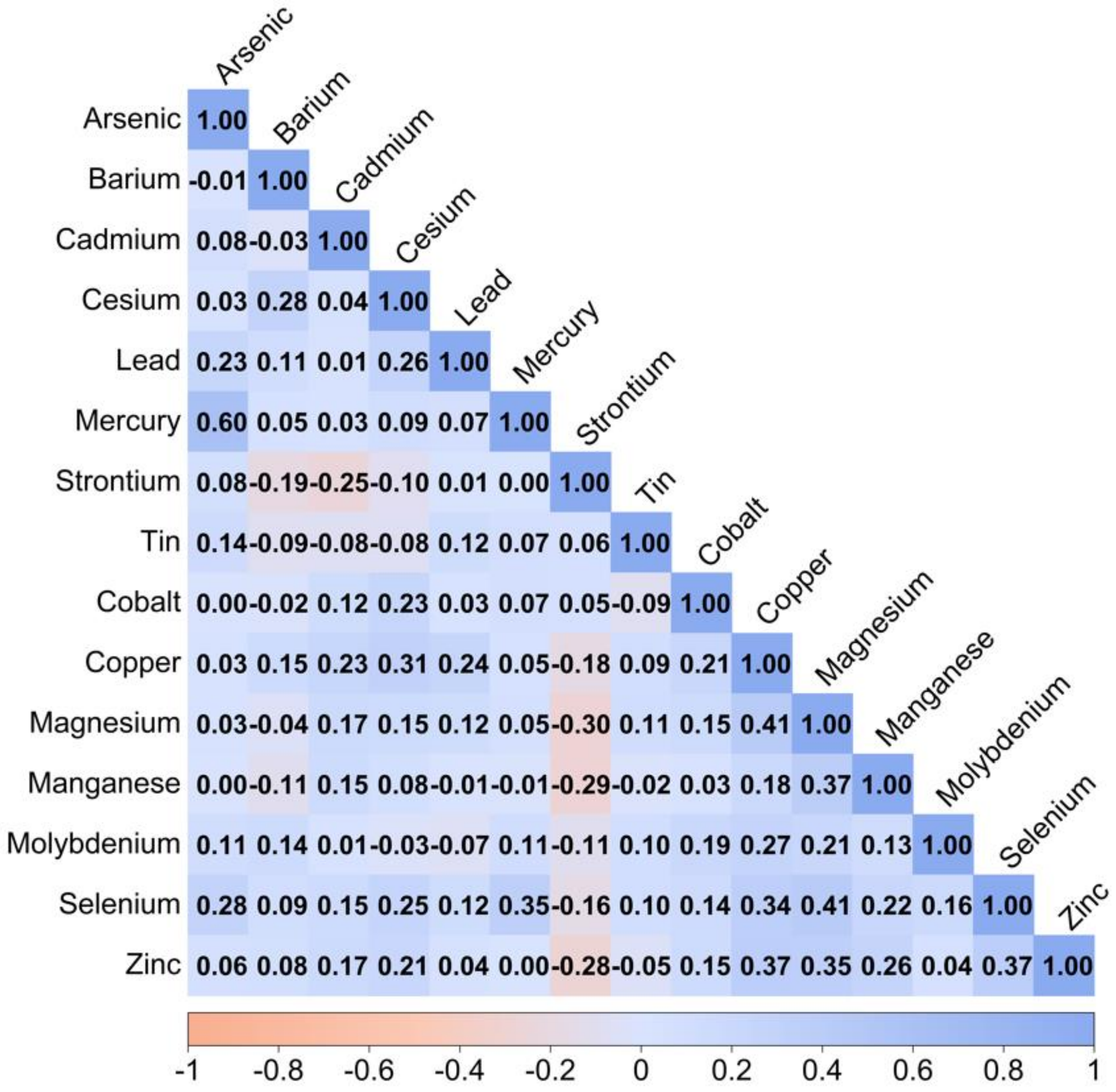
Arsenic
Barium
Cadmium
Cesium
Lead
Mercury
Strontium
Tin
Cobalt
Copper
Magnesium
Manganese
Molybdenium
Selenium
Zinc
Arsenic 1.00
Barium -0.01 1.00
Cadmium 0.08 -0.03 1.00
Cesium 0.03 0.28 0.04 1.00
Lead 0.23 0.11 0.01 0.26 1.00
Mercury 0.60 0.05 0.03 0.09 0.07 1.00
Strontium 0.08 -0.19 -0.25 -0.10 0.01 0.00 1.00
Tin 0.14 -0.09 -0.08 -0.08 0.12 0.07 0.06 1.00
Cobalt 0.00 -0.02 0.12 0.23 0.03 0.07 0.05 -0.09 1.00
Copper 0.03 0.15 0.23 0.31 0.24 0.05 -0.18 0.09 0.21 1.00
Magnesium 0.03 -0.04 0.17 0.15 0.12 0.05 -0.30 0.11 0.15 0.41 1.00
Manganese 0.00 -0.11 0.15 0.08 -0.01 -0.01 -0.29 -0.02 0.03 0.18 0.37 1.00
Molybdenium 0.11 0.14 0.01 -0.03 -0.07 0.11 -0.11 0.10 0.19 0.27 0.21 0.13 1.00
Selenium 0.28 0.09 0.15 0.25 0.12 0.35 -0.16 0.10 0.14 0.34 0.41 0.22 0.16 1.00
Zinc 0.06 0.08 0.17 0.21 0.04 0.00 -0.28 -0.05 0.15 0.37 0.35 0.26 0.04 0.37 1.00
-1 -0.8 -0.6 -0.4 -0.2 0 0.2 0.4 0.6 0.8 1

B

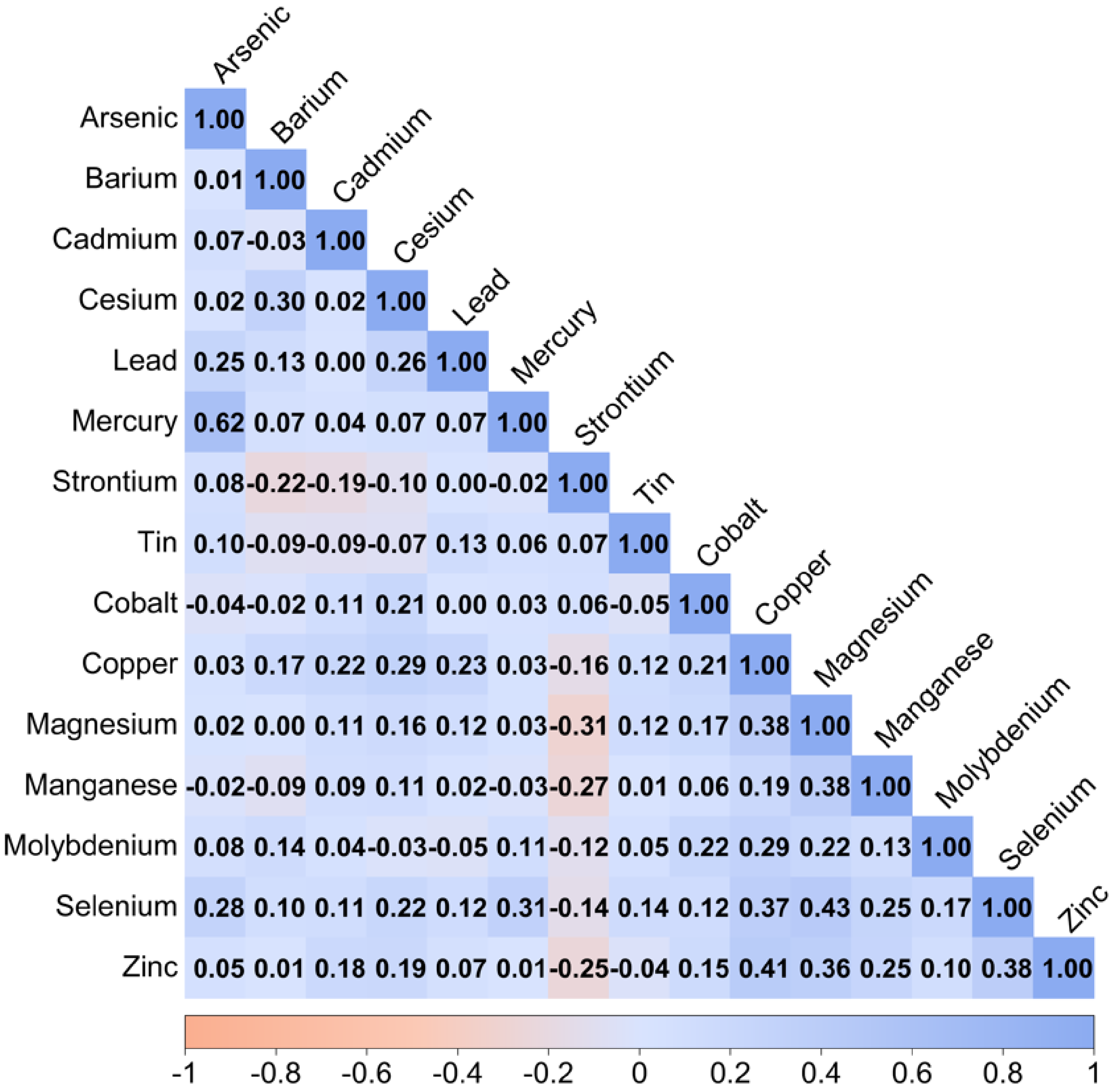

Figure S4. Spearman correlation coefficients among early childhood metal measurements in participants with at least one antibody titer outcome, and **(A)** a complete MMR (*n*=185) or **(B)** (*n*=179) a complete DTaP vaccine series.
